# Acute Encephalitis with Atypical Presentation of Rubella in Family Cluster, India

**DOI:** 10.3201/eid2410.180053

**Published:** 2018-10

**Authors:** Sumit D. Bharadwaj, Rima R. Sahay, Pragya D. Yadav, Sara Dhanawade, Atanu Basu, Virendra K. Meena, Suji George, Rekha Damle, Gajanan N. Sapkal

**Affiliations:** National Institute of Virology, Pune, India (S.D. Bharadwaj, R.R. Sahay, P.D. Yadav, A. Basu, V.K. Meena, S. George, R. Damle, G.N. Sapkal);; Bharati Vidyapeeth Deemed University Medical College and Hospital, Sangli, India (S. Dhanawade)

**Keywords:** acute encephalitis, atypical, rash, rubella, next-generation sequencing, viruses, India, meningitis/encephalitis

## Abstract

We report 3 atypical rubella cases in a family cluster in India. The index case-patient showed only mild febrile illness, whereas the other 2 patients showed acute encephalitis and died of the disease. We confirmed rubella in the index and third cases using next-generation sequencing and IgM.

Rubella is usually considered a mild viral infection. Approximately 25%–50% of infections are asymptomatic (*1*). Differential diagnosis of viral acute encephalitis syndrome (AES) caused by rubella, herpes simplex virus, measles, varicella zoster virus, and Epstein–Barr virus infections can be accomplished through the unique presentation of rash in each case. Rubella typically presents as fever with rash and is mostly diagnosed clinically, but rubella leading to fatal AES is rare (1/6,000 cases) (*2*). A cluster of rubella-associated encephalitis has been reported from Japan (*3*) and rubella encephalitis without rash from Tunisia (*4*).

We report on rubella in 3 unvaccinated siblings in India. We investigated this family cluster at the request of the treating physician in August 2017, on the eighth day postinfection of the index case-patient.

## The Study

The affected family belonged to a lower-middle income group in a village in Maharashtra, India. The father worked as a ward attendant at a hospital, and the mother was a homemaker. There were 3 children in the family; the index rubella case-patient was a 7-year-old girl, who recovered following a mild febrile illness. The 2 other siblings, an 8-year-old girl and a 2-year-old boy, died of acute encephalitis within 4 days of onset of disease (Figure). No history of similar illness in the neighborhood, travel history, or visitors were reported. There was also no history of consumption of fruits or accidental ingestion of any toxic drugs or pesticides before symptom onset. Nutritional status for all children was normal for height, weight, and body mass index.

**Figure:**
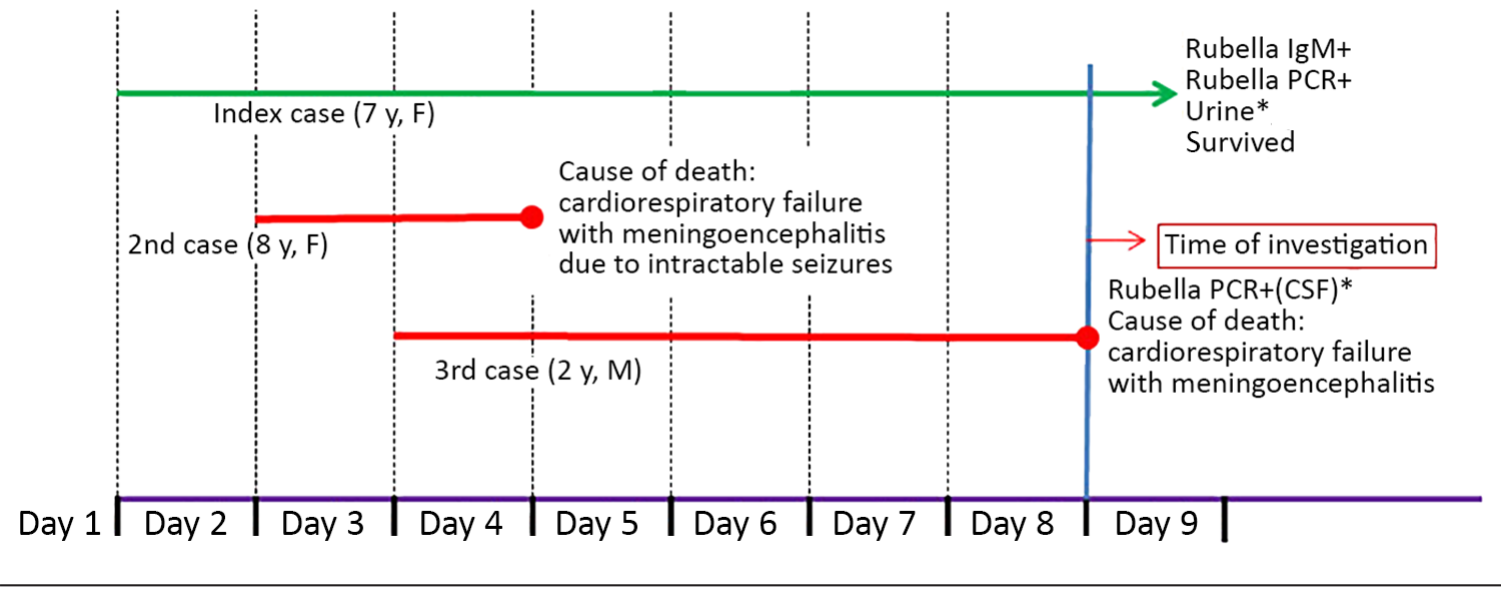
Timeline of clinical events for 3 siblings infected with rubella and encephalitis, India. *Negative for Japanese encephalitis virus, Chandipura virus, dengue virus, West Nile virus, enterovirus, and herpes simplex virus. CSF, cerebrospinal fluid.

The index case-patient developed a low-grade intermittent fever; her temperature rose in the evening. The fever subsided within 2 days after the patient took acetaminophen. She had not been immunized against rubella. Her throat was uncongested and there were no rashes or mucocutaneous lesions. Systematic examination revealed no abnormalities. Biochemical tests were normal. Tests for dengue virus and malarial parasites were negative. The child recovered without any sequelae. The investigating team examined her on the eighth day following onset of symptoms.

Two days following onset of illness in the index case-patient, her 8-year-old sister developed a high-grade fever and eye pain. There was no history of rash or mucocutaneous lesions. On day 2 of her illness, she had multiple episodes of convulsions with nonprojectile vomiting and was admitted to a hospital. On day 3, she was put on a mechanical ventilator, but she died that day. The cause of death was cardiorespiratory failure with meningoencephalitis due to intractable seizures. This child had not been immunized for rubella; no clinical samples were available to test.

One day after the onset of fever in the second patient, her 2-year-old brother developed a mild fever with no rash or mucocutaneous lesions. His intermittent fever subsided after he took acetaminophen. Two days later, he developed a high-grade fever with convulsions and nonprojectile vomiting. He became semiconscious, with decerebrated rigidity. He became comatose and was put on mechanical ventilation on day 4 of his illness. Plantar reflex was absent, and deep tendon reflexes were diminished. No cardiovascular system abnormality was noted. His chest was clear, with no wheeze or stridor. Abdominal examination revealed no notable organomegaly. The child received antimicrobial drugs, phenytoin, mannitol, and acyclovir. He died on day 5 of his illness. Details from the medical records for the second and third case-patients are provided in Technical Appendix 1 Table 1.

We tested serum and urine samples from the index case-patient and serum, urine, and cerebrospinal fluid (CSF) samples from the third case-patient for Japanese encephalitis virus (*5*), West Nile virus (*6*), Chandipura virus (*7*), and enteroviruses (*8*) using real-time quantitative reverse transcription PCR (qRT-PCR); for dengue, chikungunya, and Zika viruses using CDC Trioplex qRT-PCR; and for rubella using RT-PCR (*9*). We further used quantitative PCR to test for varicella zoster virus DNA (*10*) and PCR for herpes simplex virus 1 (Genekam Biotechnology, Duisburg, Germany, No. K091). We also tested samples for rubella IgM and IgG using a Siemens Healthcare Kit (Siemens, Erlangen, Germany). We extracted RNA and DNA using a QIAAmp total nucleic acid extraction kit (QIAGEN, Hilden, Germany).

We obtained a rubella nested PCR product of 185 bases from a CSF sample from the third case-patient; no amplification was obtained from serum and urine, so we used the CSF sample for next-generation sequencing (*11*)*.*We tested serum samples from close contacts of the index and third case-patients for the presence of rubella IgM and IgG (Table 1; Technical Appendix 2). We prepared the RNA library following the defined protocol (*12*) using a TruSeq Stranded mRNA Library preparation kit (Illumina, San Diego, CA, USA); quantified it using a KAPA Library Quantification Kit (Roche, Indianapolis, IN, USA); and loaded it on an Illumina Miniseq NGS platform. We imported raw RNA data of 147 MB in CLC Genomics Workbench software (QIAGEN) for further analysis. We assembled the RNA data using de novo and reference assembly methods and obtained 1.9 million reads with an average length of 138 bp. From the total read, 0.57% mapped to the reference genome (GenBank accession no. JN635296) with an average length of 123 bp. De novo assembly of reads gave 86 contigs with an average length of 1,281 bp. Reference mapping led to a ≈8 kb region (4.5 kb of nonstructural protein [NSP], 3 kb of structural protein [SP], and 0.5 kb in both NSP and SP regions) that was broken intermittently (GenBank accession nos. MG987207.1 for NSP and MG987208.1 for SP) (Technical Appendix 1 Figure 1). We used a 732-bp E1 gene sequence to identify the rubella genotype (Technical Appendix 1 Figure 1). The phylogenetic tree that we constructed revealed that this virus belongs to rubella genotype 2B (Table 2; Technical Appendix 1 Figure 2).

**Table 1:** Laboratory investigations and results of the clinical samples of close contacts of case-patients with rubella and encephalitis in a family cluster in India

NIV no.*	Specimen	Contact relationship to index and third case-patients	Age, y/sex	Contact with case-patients	Anti-rubella IgM ELISA	Anti-rubella IgG ELISA
1730348	Serum	Father	40/M	All 3	Equivocal	Positive
1730348-2	Serum	Father	40/M	All 3	Equivocal	Positive
1730358	Serum	Mother	27/F	All 3	Negative	Positive
1730358-2	Serum	Mother	27/F	All 3	Negative	Positive
1730362	Serum	Physician	38/F	Index, 3rd	Negative	Positive
1730365	Serum	Physician	32/F	Index, 3rd	Negative	Positive
1730367	Serum	Physician	25/F	Index, 3rd	Negative	Positive
1730371	Serum	Physician	26/F	Index, 3rd	Negative	Positive
1730382	Serum	Physician	26/F	Index, 3rd	Negative	Positive
1730411	Serum	Physician	25/F	Index, 3rd	Negative	Positive
1730389	Serum	Physician	26/F	Index, 3rd	Negative	Positive
1730391	Serum	Nursing staff	22/F	Index, 3rd	Positive	Positive
1730394	Serum	Nursing staff	25/F	Index, 3rd	Negative	Positive
1730396	Serum	Nursing staff	29/F	Index, 3rd	Negative	Equivocal
1730398	Serum	Paternal uncle 1	33/M	Index, 3rd	Negative	Positive
1730374	Serum	Paternal aunt 1	25/F	Index, 3rd	Negative	Positive
1730933	Serum	Paternal uncle 2	34/M	Index, 3rd	Negative	Positive
1730936	Serum	Cousin	11/M	Index, 3rd	Negative	Positive
1730939	Serum	Cousin	8/M	Index, 3rd	Negative	Negative
1730941	Serum	Paternal aunt 2	21/F	Index, 3rd	Negative	Positive
1730944	Serum	Grandfather	70/M	Index, 3rd	Negative	Positive
1730948	Serum	Grandmother	65/F	Index, 3rd	Negative	Positive
1730950	Serum	Paternal aunt 2	26/F	Index, 3rd	Negative	Positive
*NIV, National Institue of Virology.							

**Table 2:** Rubella virus variability with respect to Indian strain BAS*†

Genotype	Intragroup variability, %	
	Nucleotide	Amino acid
GI	6–9	1–2
GII	2–8	0–1
GIIA	8	0–1
GIIB	2–3	0–1
GIIC	7	0–2
NIV E1 gene	3	1

*GenBank accession no. AF039134. †NIV, National Institue of Virology.

## Conclusions

Rubella encephalitis without identification of typical rubella rash is rarely reported. In the cluster we describe, the parents were asymptomatic and positive for anti-rubella IgG when tested on the eighth day from the onset of symptoms in the index case-patient. The rubella IgM equivocal status of the father suggests that he could be a possible source of infection to the family. His occupation as a hospital ward attendant also indicates a possibility of infection either through respiratory secretions or by contact with an infectious agent on his body or on items carried to and from his workplace. In rubella, neurologic symptoms most often occur 1–6 days after the onset of the exanthem (*13*). In this cluster, rapid disease progression meant the second and third case-patients died within 4 days of illness onset. The index case-patient tested positive for rubella IgM on the eighth day postinfection and the third case-patient tested positive for rubella IgM on the fifth day postinfection, but their specimens were negative for IgG, suggesting that the children were not immunized and had not had any past rubella infection. A serum sample from the index case-patient from the 14th day postinfection was IgG positive. Through epidemiologic linkage, the causative agent in the second case may be similar to that for the other cases.

In conclusion, rubella encephalitis can present without rash. Rubella virus infection should be considered in the differential diagnosis of AES in unvaccinated children.

Technical Appendix 1 Patient information for patients infected with rubella and encephalitis, India, and mapping of the rubella virus in India.

Technical Appendix 2 Information about laboratory tests for 3 patients infected with rubella and encephalitis, India.
