# Supplementary material for: Acute Encephalitis with Atypical Presentation of Rubella in Family Cluster, India
Source: Emerg Infect Dis. 2018 Oct;24(10):1923–5. doi: 10.3201/eid2410.180053 (PMC6154155; doi:10.3201/eid2410.180053)
Supplement: Technical Appendix 1 — Patient information for patients infected with rubella and encephalitis, India, and mapping of the rubella virus in India. [file 18-0053-Techapp-s1.pdf]

Article DOI: https://doi.org/10.3201/eid2410.180053

Acute Encephalitis with Atypical Presentation of Rubella in Family Cluster, India

Technical Appendix 1

Technical Appendix 1 Table. Medical records, including hematological and biochemical parameters, for second and third case-patients on day 2 postinfection with rubella and encephalitis, India

Case-patient	Value	Reference range
Case-patient 2		
Renal function test		
Blood urea nitrogen	23 mg/dL	17–49 mg/dL
Serum creatinine	0.6 mg/dl	0.52–1.04 mg/dL
Serum sodium	137 mEq/L	135–146 mEq/L
Serum potassium	4.4 mEq/L	3.5–5.5 mEq/L
Serum chloride	105 mEq/L	95–112 mEq/L
Serum calcium	9.4 mg/dL	8–11 mg/dL
Complete blood count		
Hemoglobin	11.9 g/dL	12–15 g/dL
Leukocytes	11,700/μL (P 72, L 22)*	4,000–11,000/μL
Platelets	129,000/μL	150,000–450,000/μL
Erythrocyte morphology	Hypochromia	Normocytic, normochromic
Other tests		
C-reactive protein	9.8 mg/dL	1–3 mg/dL
Serum glutamic pyruvic transaminase (SGPT)	84.4 U/L	5–40 U/L
Widal test O-antigen	1:160	Titer <1:160
H-antigen	1:80	Titer <1:160
Malarial parasites	Negative	
Case-patient 3		
Liver function test		
Total bilirubin	0.4 mg/dL	0.1–1.2 mg/dL
Bilirubin direct	0.2 mg/dL	≤0.3 mg/dL
Bilirubin indirect	0.2 mg/dL	0.2–1.2 mg/dL
Serum albumin	3.6 g/dL	3–5 g/dL
Serum globulin	2.9 g/dL	2–3.5 g/dL
Serum glutamic oxaliacetic transaminase (SGOT)	66 U/L	5–40 U/L
Alakaline phosphatase (ALT)	123 units/L	45–115 U/L
Renal function test		
Blood urea nitrogen	23 mg/dL	17–49 mg/dL
Serum creatine	0.6 mg/dL	0.52–1.04 mg/dL
Serum sodium	142 mEq/L	135–146 mEq/L
Serum potassium	3.6 mEq/L	3.5–5.5 mEq/L
Serum calcium	8.0 mg/dL	8–11 mg/dL
Serum ionic calcium	1.1 mg/dL	4.64–5.28 mg/dL
Complete blood count		
Hemoglobin	9.3 g/dL	12–15 g/dL
Leukocytes	5,800/μL (P 63, L 29)	4,000–11,000/μL
Erythrocyte morphology	Hypochromia, microcytosis, anisocytosis, poikilocytosis	Noromocytic, normochromic
Platelets	228,000/μL	150,000–450,000/μL
Cerebrospinal fluid examination		
Leukocytes	10 cells/μL	0–5 cells/μL
Polymorphs	0	< 2 cells/μL
Lymphocytes	100%	<70%
Proteins	383 mg/dL	15–45 mg/dL

Case-patient	Value	Reference range
Glucose	210 mg/dL	50–80 mg/dL
Arterial blood gas analysis		
pH	7.41	7.35–7.45
pCO_2	25.7mmHg	35–45 mmHg
pO_2	193.2 mmHg	135–148 mmHg
HCO_3	18.5 mmol/L	22–26 mmol/L
Other tests		
Blood glucose level	107 mg/dL	80–130 mg/dL
Serum lactate	21 mg/dL	5–15 mg/dL
Widal test: O antigen	1:40	Titer <1:160
H antigen	1:40	Titer <1:160
Urine routine and microscopy	Within normal limits	
Blood culture (48 h)	No growth/sterile	
Chest X-ray	Within normal limits	
Computed tomography, brain	Within normal limits	
Electroencephalogram	Generalized epileptiform discharges with occasional delta burst, suggestive of epileptic encephalopathy	
Fundus examination	No papilloedema	

*L, lymphocytes; P, polymorphs

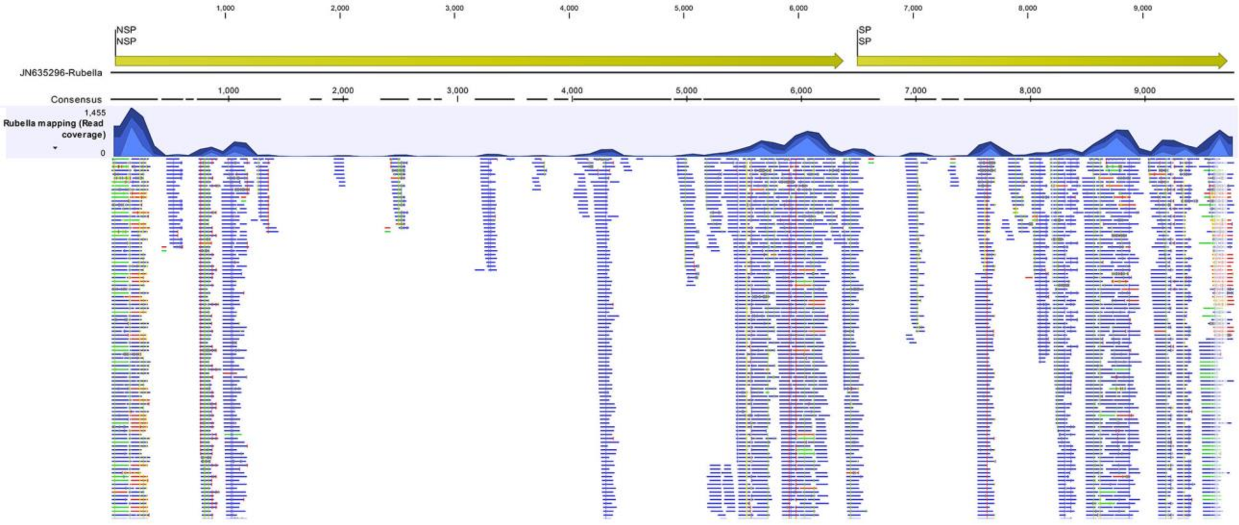

Technical Appendix 1 Figure 1. Reference mapping of Indian rubella virus.

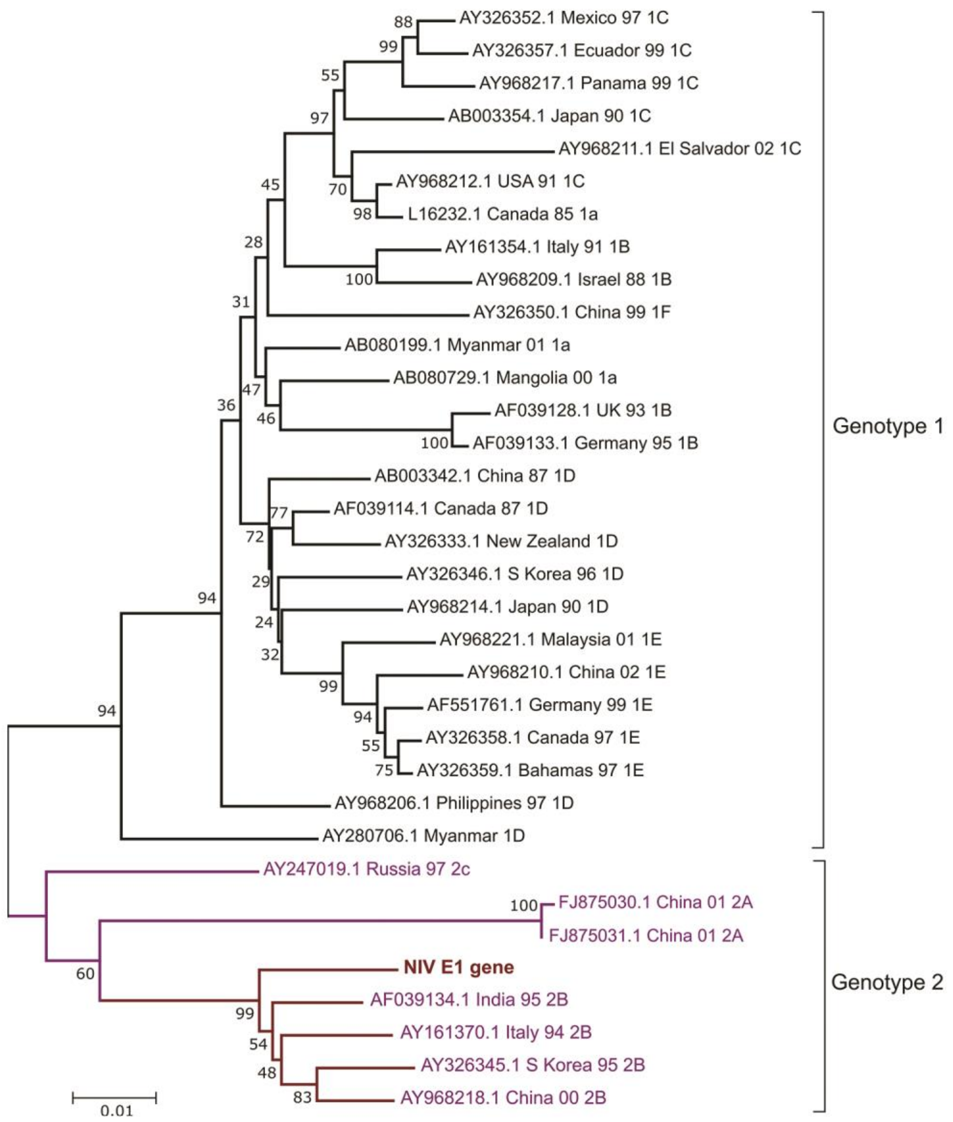

Technical Appendix 1 Figure 2. Phylogenetic tree of the E1 gene (732 bp) obtained from CSF sample using the Tamura-Nei method, with bootstrap replication of 500. Scale bar indicates nucleotide substitutions per site.
